# Lower urinary tract symptoms and hematuria in Rheumatoid arthritis (LUTH-RA) study

**DOI:** 10.12669/pjms.41.3.8316

**Published:** 2025-03

**Authors:** Abrar Ahmed Wagan, Paras

**Affiliations:** 1 Abrar Ahmed Wagan, MBBS, FCPS (Medicine), FCPS (Rheumatology), FACR Associate Professor of Rheumatology, Indus Medical College, Tando Mohammad Khan, Sindh, Pakistan; 2 Paras, MBBS, FCPS (Diagnostic Radiology) Assistant Professor, Department of Radiology, Bilawal Medical College Jamshoro, Sindh, Pakistan

**Keywords:** DAS-28, Hematuria, LUTS, Nocturia, Quality of life, Rheumatoid arthritis

## Abstract

**Objective:**

To determine the frequency of hematuria and lower urinary tract problems in rheumatoid arthritis cohort.

**Method:**

This cross sectional prospective study was conducted at department of Rheumatology Indus Medical College Tando Mohammad Khan from August 1, 2022 to March 3, 2023. Total 229 patients were selected after written and informed consent; demographic details were taken. Bristol Female Lower Urinary Tract Questionaries’ (BFLUTS) was filled and all male participants were asked for ultrasound scan of prostate and freshly voided midstream urine was collected for microscopic hematuria.

**Results:**

In this study the prevalence of Lower Urinary Tract Symptoms (LUTS) was (77.3%) and microscopic hematuria (34.5%). Major symptoms of LUTS were: nocturia (69%), bladder pain (35.4%), leaking before going to toilet (40.6%), frequency of incontinence (24.5%), nocturnal incontinence (27.9%), sex life spoiled due to urinary symptoms (22.7%), avoid situation where no toilet (30.1%) and overall interference of life (32.8%) cases. A significant association of DAS-28 with LUTS and microscopic hematuria was seen (p<0.01).

**Conclusion:**

Lower urinary tract problems and microscopic hematuria are common in both genders, and severity of RA, increases LUTS and affects quality of life.

## INTRODUCTION

Rheumatoid arthritis is an important multisystem autoimmune disease, mostly affecting females in young age, clinical manifestations of joint pain and swelling, deformities with disability and other organ involvement, adversely affects the quality of life.^1^ Although it affects rarely the urinary tract, clinically presents as genitourinary infections, renal stones, and newer symptoms of lower urinary tract.^2^ Autoimmune disease causing arthritis are associated increased residual urine in urinary bladder, acute urinary retention, and there is higher chances of stone formation in urinary system due to changes in calcium metabolism.^3^-^5^

Lower urinary tract symptoms (LUTS) is related to complains of storage, voiding, and post-urination, The direct relation between RA and LUTS is not defined yet but few studies have reported the frequency of LUTS in patients with autoimmune rheumatic diseases, especially increased prevalence of voiding dysfunction, both conditions drastically affects the patient’s quality of life (QoL).^6^ RA with secondary Sjogren’s syndrome there is higher risk of LUTS, especially of urination frequency than normal individuals.^7^

Rouached et al., found that LUTS was highly severe in rheumatic disorders than controls (p = 0.03) and no difference was between the two groups in LUTS affecting quality of life (p = 0.2).^8^ In RA who have renal dysfunction and/or urinary alterations (proteinuria and/or hematuria) mesangial glomerulonephritis is the most common histologically type postmortem studies have shown that nephrosclerosis is the most frequent kidney lesion.^9^ Annual incidence of urinary tract infection (UTI) with hospitalization was 2.09%, as against 0.97 and 0.91% for two control groups (RR = 2.16 and 2.29) and mortality and morbidity is also increased from infections of genitourinary and respiratory infections along with sepsis.^2^,^10^ In a case control study mild mesangial glomerulopathy was the most common renal biopsy finding in patients with RA with isolated hematuria, found in 13 of 15 adequate renal biopsy and the cause of hematuria remained uncertain or unknown in 52% of the patients with RA and in 61% of controls.^11^

In RA kidney involvement is variable may present primarily as different types of glomerulonephritis (GN) related to the disease process, or rarely as rheumatoid vasculitis and amyloidosis secondary to chronic inflammatory state.^12^ Till now there is no standardized validated instrument to evaluate the spectrum of LUTS across both men and women.^13^

The objective of this study was to determine the frequency of hematuria and lower urinary tract problems in rheumatoid arthritis cohort.

## METHODS

The cross sectional study was conducted during August 1, 2022 to March 1, 2023 at department of Rheumatology Indus Medical College Tando Mohammad Khan, Sindh.

### Ethical Approval:

The study was approved by the hospital Ethics Committee Ref. (51/2022, dated August 1, 2022), and written and informed consent were taken.

### Inclusion & Exclusion Criteria:

Cases of Rheumatoid Arthritis as per ACR/EULAR criteria 2010 were included, and SLE, Scleroderma, Psoriatic arthritis, Polymyositis, mixed connective tissue diseases, use of cyclophosphamide, known cases of urogenital malignancy, history of prostate surgery in males, trauma to urogenital area or any kind of pelvic surgery or radiation to pelvic area were excluded.

All study participants underwent the demographic details and checkup afterward their blood pressure, BMI were measured with standard protocol. A predefined Bristol female LUTS Questionnaire was filled and scores were noted, freshly voided urine sample was collected and sent for detection of microscopic hematuria (characterized as >3 red blood cells/high-power microscopic field,^9^ male patients underwent the Ultrasound of Prostate for Benign Prostate hypertrophy on sonographic protocol and if found they were excluded from study.

### Statistical Analysis:

Data were stored and analyzed using IBM-SPSS version 23.0; counts with percentages were reported on baseline characteristic of studied samples included age group, gender, BMI, disease duration, DAS-28. Descriptive on symptoms of LUTS were reported, association of DAS-28 was tested with LUTS, disease duration and Microscopic Hematuria. P-values less than 0.05 were considered statistically significant. Bar diagram was also used to display the significant association of DAS-28.

## RESULTS

**Fig.1:**
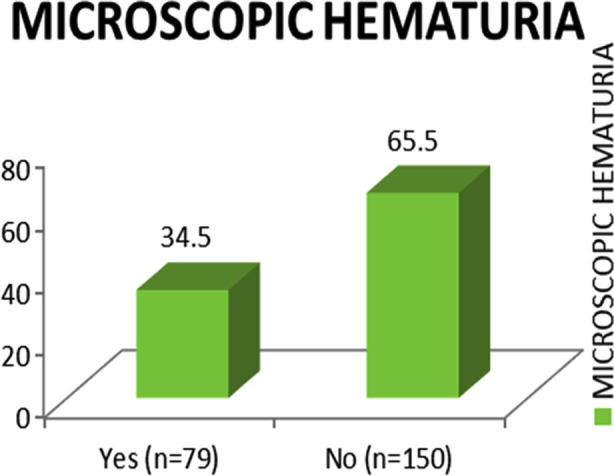
Frequency of Microscopic Hematuria.

This study cohort had, mean age 43.9 (SD=±13.3), females were (86%), mostly with >40-years age (56.8), disease duration of (<1-5) years in (43%), and moderate disease activity score in (62.9%), (Table-I). The prevalence of lower urinary tract symptoms accounted for (77.3%) and microscopic hematuria was (34.5%) Fig.1.

**Table-I:** Baseline characteristics of studied samples (n=229).

Characteristics		n	%
Age group	≤40 Years	99	43.2
	>40 Years	130	56.8
	Mean ±SD	43.9	±13.3
Sex	Female	198	86.5
	Male	31	13.5
BMI	Normal	39	17.0
	Overweight	82	35.8
	Obese	108	47.2
	Mean ±SD	28.7	±3.8
Disease duration	1 - 5 Years	100	43.7
	6 - 10 Years	92	40.2
	>10 Years	37	16.2
	Mean ±SD	7.09	±3.7
Das -28	Remission	16	7.0
	Low disease activity	52	22.7
	Moderate disease activity	144	62.9
	High disease activity	17	7.4
	Mean ±SD	3.9	±2.1

LUTS Belfast questionnaire descriptive analysis results, showed the predominant symptom from each section: Storage Domain: nocturia in (69%), Voiding domain: bladder pain in (35.4%), Incontinence domain: leaking before going to toilet in (40.6%), Sexual function domain: sex life spoiled due to urinary symptoms in (22.7%), Quality of life domain: overall interference of life in (32.8%), Table-II.

**Table-II:** Descriptive on symptoms of LUTS.

Symptoms	n	%
***Storage***		
Nocturia	158	69.0
Urgency	68	29.7
Frequency	59	25.8
***Voiding***		
Bladder pain	81	35.4
Hesitency	24	10.5
Strain to urinate	18	7.9
Intermittency	22	9.6
***Incontinence***		
Leaking before going to toilet	93	40.6
Frequency of incontinence	56	24.5
Stress incontinence	59	25.8
Unpredictable incontinence	61	26.6
Nocturnal incontinence	64	27.9
***Sexual function***		
Sex life spoiled due to uri symptom	52	22.7
Leaking during intercourse	51	22.3
***Quality of life***		
Change outer clothings	53	23.1
Cut down fluids	62	27.1
Affects daily life	63	27.5
Avoid situation where no toilet	69	30.1
Overall interference of life	75	32.8

Association of disease severity (DAS-28) with LUTS, disease duration and microscopic hematuria, shows as increase in RA severity is linked to enhanced occurrence of lower urinary tract symptoms (<0.01) and microscopic hematuria (<0.01), (Table-III).

**Table-III:** Association of DAS-28 with LUTS, disease duration and microscopic hematuria.

Parameters		DAS -28												P-value
		Remission			Low disease activity			Moderate disease activity			High disease activity			
		n	%	n		%	n		%	n		%		
Lower Urinary Tract Symptoms	Yes	8	50.0	34		65.4	118		81.9	17		100.0	<0.01*	
	No	8	50.0	18		34.6	26		18.1	0		0.0
Disease Duration	1 - 5 Years	8	50.0	25		48.1	60		41.7	7		41.2	0.64	
	6 - 10 Years	6	37.5	23		44.2	56		38.9	7		41.2
	>10 Years	2	12.5	4		7.7	28		19.4	3		17.6
Microscopic Hematuria	Yes	1	6.3	12		23.1	57		39.6	9		52.9	<0.01*	
	No	15	93.8	40		76.9	87		60.4	8		47.1

* P<0.05 was considered statistically significant using Pearson Chi Square test

## DISCUSSION

In our study we found LUTS prevalence was (77.3%) and microscopic hematuria (34%) and moderate to high disease activity was significantly associated with LUTS and hematuria (p-0.05), in-contrast to our findings, Faris A et al, in a study of 89 patients with only LUTS evaluation but not hematuria, found the prevalence of 94% and didn’t report any significant association between LUTS and disease activity.^6^

In current study (44%) cases had sexual life affected due to urinary problems while other study has reported zero prevalence. Immuno-suppressants medicines used for various Autoimmune diseases, suppress immunity like glucocorticoids, methotrexate, mycophenolate, azathioprine, hydroxychloroquine and monoclonal antibodies to various cytokines (e.g., tumor necrosis factor, interleukins 1 and 6) and cell receptors (anti-CD20) may have varying suppressive effects on cell-mediated and humoral immunity.^14^ The severity and the prevalence of lower urinary tract symptoms and overactive bladder has increased in systemic lupus erythematosus, rheumatoid arthritis and sjogren’s syndrome.^15^ Makino H et al, in 100 ambulatory patients with RA, found that urinary problems like proteinuria and microscopic hematuria were seen in one third of cases.^16^

Various studies have suggested that urinary tract infections caused by Proteus spp. have a key role in the etiopathogenesis of RA, in nationwide studies from different countries in RA patients these are the most prevalent urinary pathogen than general population of the same country where it comes at fourth number.^17^ In another study with100 RA patients, 41 (41%) had UTI with positive urine culture for proteus mirabilis, and 59 (59%) had different organisms.^18^ In overlap syndrome (RA and vasculitis), in 246 patients ANCAs antibodies were found in 21% (n = 52), these were associated with disease activity and severity and acts as independent predictor renal pathology.^19^ Proteinuria and/or hematuria in patients with RA will warn for glomerular lesions, early diagnosis and treatment can help preserve kidney functions.^20^

In a case report RA caused complete urinary tract wall inflammation with thickening leading to hydronephrosis, hematuria, and post-renal AKI, histopathology of vesicular trigone showed chronic inflammation, which persisted despite antibiotics usage but responded to corticosteroids and immunosuppressive therapy.^21^ Norihiro N et al. reported a case of RA with urethritis presented as recurrent massive hematuria, histopathology showed a chronic inflammatory changes leading to stenosing ureteritis.^22^

Another study showed that in a middle-aged or young adult female with RA MPO ANCA- associated crescentic glomerulonephritis with progressive degradation in renal function, limited extra-renal symptoms and micro-hematuria. Histopathology revealed advanced chronic damage with glomerular sclerosis and less development of crescent. MPO-ANCA associated crescentic GN in RA tended to demonstrate gradual progressive renal insufficiency with minor extra-renal manifestations.^9^ Apart from the urinary tract problems there are problems with renal system functioning as well Abrar et al, reported prevalence of Impaired renal functions of 14.6% (n=38) in RA cohort.^23^

### Limitations:

This is small scale cross sectional study, results can’t be generalized, but this is first study about the urinary complains in local populations and earlier none of the studies has ventured to microscopic hematuria in RA and this study paves the way for further studies about similar topics.

## CONCLUSION

Lower urinary tract problems are a common complication of RA and significantly impact a person’s quality of life and may require urgent care. Patients should speak with their rheumatologist to discuss these issues and treatment options, leading to significant improvement in their symptoms and quality of life.

### Authors’ Contribution:

**AAW:** Design, drafting, data acquisition data analysis, data interpretation, final approval and responsible for the accuracy of the study. **PS**: Data acquisition, data analysis, interpretation, drafting, final approval.
